# Case Report: Multiple Sclerosis Relapses After Vaccination Against SARS-CoV2: A Series of Clinical Cases

**DOI:** 10.3389/fneur.2021.765954

**Published:** 2021-10-22

**Authors:** Riccardo Nistri, Elena Barbuti, Virginia Rinaldi, Laura Tufano, Valeria Pozzilli, Antonio Ianniello, Fabiana Marinelli, Giovanna De Luca, Luca Prosperini, Valentina Tomassini, Carlo Pozzilli

**Affiliations:** ^1^Neurology Unit, Sant'Andrea Hospital, Sapienza University, Rome, Italy; ^2^Institute of Advanced Biomedical Technologies (ITAB), Department of Neurosciences, Imaging and Clinical Sciences, University G. d'Annunzio of Chieti-Pescara, Chieti, Italy; ^3^MS Centre, Department of Clinical Neurology, SS. Annunziata University Hospital, Chieti, Italy; ^4^MS Centre, Sant'Andrea Hospital, Sapienza University, Rome, Italy; ^5^MS Centre, Department of Neurology, Fabrizio Spaziani Hospital, Frosinone, Italy; ^6^MS Centre, Department of Neurosciences, S. Camillo-Forlanini Hospital, Rome, Italy

**Keywords:** SARS-CoV2 infection, COVID-19 vaccine, multiple sclerosis relapse, MRI activity, lesions, adverse event

## Abstract

**Objective:** To describe a temporal association between COVID-19 vaccine administration and multiple sclerosis (MS) relapses.

**Methods:** This case series study was collected in four MS Centres in Central Italy, using data from 16 MS patients who received COVID-19 vaccination and presented both clinically and radiologically confirmed relapses between March and June 2021. We collected patients' relevant medical history, including demographics, MS clinical course, disease-modifying treatment (DMT) received (if applicable), and data from MRI scans obtained after the COVID-19 vaccination.

**Results:** Three out of 16 patients received a diagnosis of MS with a first episode occurring after COVID-19 vaccination; 13 had already a diagnosis of MS and, among them, 9 were on treatment with DMTs. Ten patients received BNT162b2/Pfizer-BioNTech, 2 patients mRNA-1273/Moderna, and 4 patients ChAdOx1 nCoV-19/AstraZeneca. All MS relapses occurred from 3 days to 3 weeks after receiving the first dose of the COVID-19 vaccination or the booster. All patients had evidence of radiological activity on MRI.

**Discussion:** Clinical and radiological findings in these cohort of MS patients confirmed disease re/activation and suggested a temporal association between disease activity and COVID-19 vaccination. The nature of this temporal association, whether causative or incidental, remains to be established.

## Introduction

Patients with multiple sclerosis (MS) have an increased risk of respiratory infections, especially patients presenting severe disability and on disease-modifying treatments (DMTs) (1). Infections can trigger MS relapses (2), and thus, vaccination in MS patients should be pursued as a general policy in order to reduce the risk of infections (3). Despite the long-standing debate over an increased risk of relapse occurrence after vaccination, the existence of this phenomenon has not been confirmed (4).

The ongoing coronavirus pandemic led to an unprecedented vaccination campaign that included MS patients. In Italy, two types of vaccines were available: (i) mRNA-vaccines (BNT162b2 Pfizer/BioNTech and mRNA-1273 Moderna) (5); (ii) adenovirus-vectored vaccine (ChsdOx1 nCoV-19, AZD12222, AstraZeneca) (6).

Here, we describe 16 cases of clinically and radiologically confirmed MS re/activation that occurred after the administration of COVID-19 vaccines in MS patients regularly followed in four MS Centres in Central Italy from March to June 2021 (Table 1).

**Table 1:** Demographic and clinical baseline characteristics of the MS patients.

**No. cases**	**Age**	**Sex**	**EDSS**	**DMT**	**Year of last relapse**	**Disease duration**	**Type of vaccine**	**Dose**	**Time of symptom onset after vaccine**	**Steroids use**	**No. new MRI lesions**	**Timing of MRI after symptom onset**
1	45	M	2.5	Ocrelizumab	2020	9 years	ChAdOx1 nCoV-19	1	21 days	Yes	2 brain Gd-, 1 spine Gd-	50 days
2	48	F	2.0	None	New diagnosis	ND	ChAdOx1 nCoV-19	1	8 days	Yes	1 brain Gd+	18 days
3	54	F	2.5	None	2014	28 years	ChAdOx1 nCoV-19	1	3 days	No	1 spine Gd+	17 days
4	66	F	2.5	None	New diagnosis	ND	ChAdOx1 nCoV-19	1	7 days	Yes	4 brain Gd+	17 days
5	42	f	4.0	Ocrelizumab	2019	2 years	mRNA-1273	1	14 days	No	1 brain Gd+	17 days
6	57	F	6.0	None	2015	20 years	mRNA-1273	2	14 days	Yes	1 brain Gd+	13 days
7	49	F	1.5	DMF	2013	8 years	BNT162b2/Pfizer-BioNTech	1	5 days	Yes	1 brain Gd+, 1 spine Gd+	7 days
8	39	M	2.0	DMF	2018	7 years	BNT162b2/Pfizer-BioNTech	1	10 days	Yes	2 brain Gd+, 1 spine Gd-	7 days
9	39	F	1.0	None	new diagnosis	ND	BNT162b2/Pfizer-BioNTech	1	3 days	Yes	1 brain Gd+	9 days
10	60	F	3.5	DMF	2014	23 years	BNT162b2/Pfizer-BioNTech	1	2 days	No	1 brain Gd+	3 days
11	30	F	1.5	Cladribine	2020	3 years	BNT162b2/Pfizer-BioNTech	2	20 days	Yes	2 brain Gd+	36 days
12	58	F	5.0	None	2018	21 years	BNT162b2/Pfizer-BioNTech	1	3 days	Yes	1 brain ring Gd+	38 days
13	34	F	2.5	None	2021	3 months	BNT162b2/Pfizer-BioNTech	2	4 days	Yes	3 brain Gd+, 1 spine Gd-	16 days
14	35	F	2.0	DMF	2019	16 years	BNT162b2/Pfizer-BioNTech	2	1 day	Yes	3 brain Gd+	13 days
15	54	M	2.0	Teriflunomide	2020	18 years	BNT162b2/Pfizer-BioNTech	1	7 days	Yes	2 brain Gd+	4 days
16	37	M	1.5	DMF	2019	2 years	BNT162b2/Pfizer-BioNTech	2	10 days	Yes	1 brain Gd+	9 days

*No., number of; EDSS, expanded disability status scale; M, male; F, female; DMT, disease modifying treatment; DMF, dimethyl fumarate; ND, new diagnosis; Gd, gadolinium-enhancing lesion; MRI, magnetic resonance imaging*.

## Case Series

### Case 1

**Figure 1:**
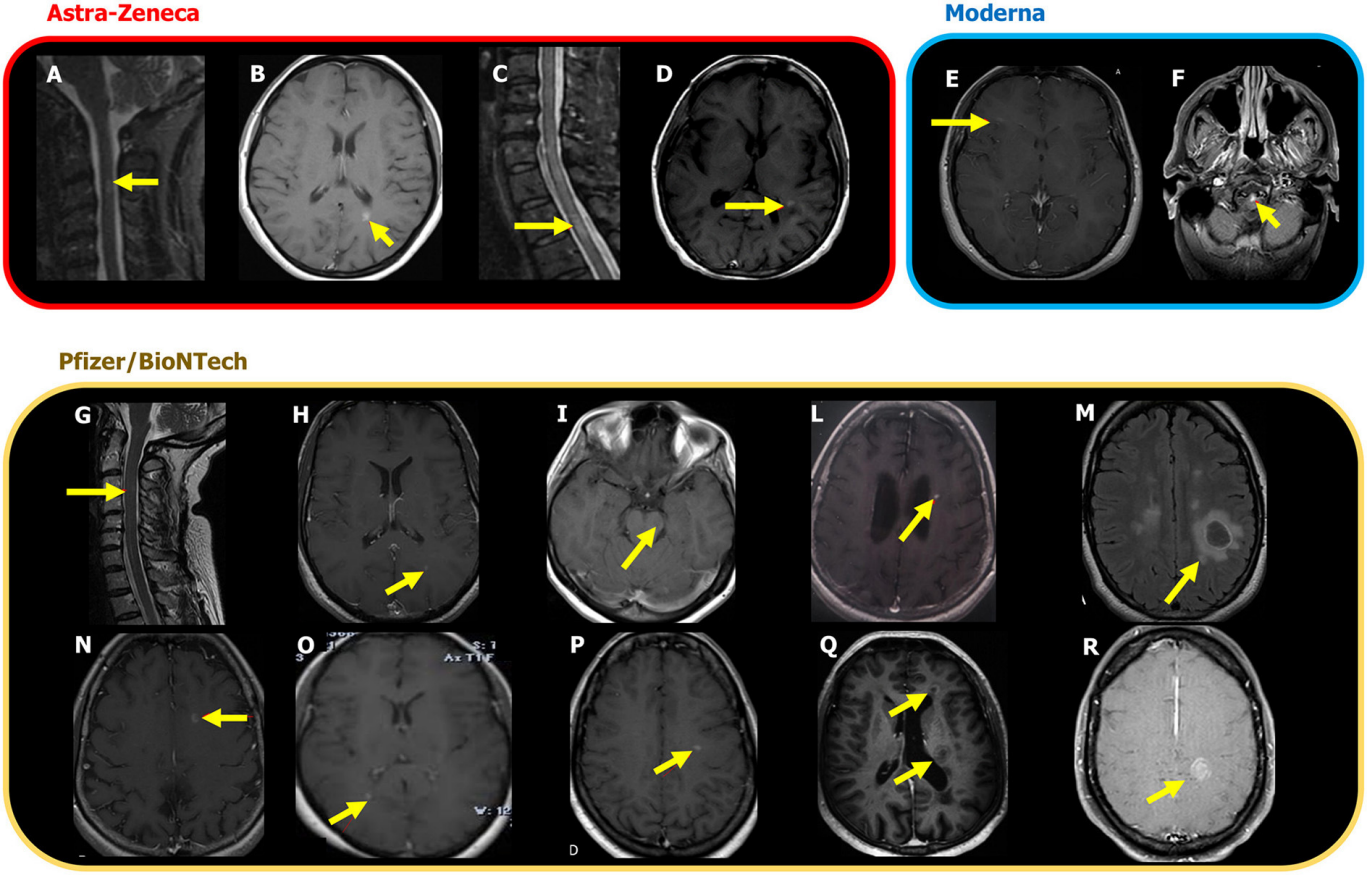
New MRI lesions associated with the MS episodes occurred after ChAdOx1 nCoV-19 (AZD12222), mRNA-1273, Moderna and BNT162b2, Pfizer/BioNTech vaccine. The lesions are shown on T2 weighted images or on post-contrast T1 weighted images and are indicated by yellow arrows. **(A)** Case 1: C3 lesion; **(B)** Case 2: new enhancing lesion in corpus callosum and multiple white matter unenhanced lesions in periventricular areas and in the mesial occipital lobe; **(C)** Case 3: new enhancing lesion in the thoracic cord; **(D)** Case 4: multiple hyperintense lesions in the supra and infratentorial white matter, four of which are with contrast enhancement; **(E)** Case 5: new brain lesion with contrast enhancement. **(F)** Case 6: enhancing bulbar lesion; **(G)** Case 7: C3 lesion with contrast enhancement; **(H)** Case 8: new brain enhancing lesion; **(I)** Case 9: a new contrast enhancing lesion in the mesencephalon. **(L)** Case 10: new enhancing brain lesion. **(M)** Case 11: enhancing brain lesion with conspicuous oedema; **(N)** Case 12: a new active lesion with ring enhancement in the left frontal white matter. **(O)** Case 13: three new brain enhancing lesions, one of which is indicated by the arrow; **(P)** Case 14: three new enhancing lesions in the left temporal lobe and one, indicated here, in the left centrum semiovale; **(Q)** Case 15: two ring-enhancing lesions localized in the white matter adjacent to the left frontal horn and in the left middle periventricular region. **(R)**. Case 16: new enhancing lesions, one of which is tumefactive, localized in the white matter of the left centrum semiovale.

A 45-year-old man received a diagnosis of MS (7) in 2012 and was started on teriflunomide and then from April 2020 with Ocrelizumab with radiological and clinical stability, as confirmed in November 2020. He received his first ChAdOx1 nCoV-19 on February 19, 2021. He experienced dysesthesia in both legs 3 weeks later. He underwent a scan on April 30, 2021 which showed two new lesions in the temporal gyri and a new spinal cord lesion at T3 level (Figure 1A).

### Case 2

A 48-year-old woman received on March 5 her first dose of ChAdOx1 nCoV-19. 8 days later, she developed visual acuity deficit from her right eye. She underwent MRI scan on March 31, where an enhancing lesion in the corpus callosum, multiple white matter unenhanced lesions, and lesions in the occipital lobe were detected (Figure 1B). Diagnosis of MS was made, and she was treated with high dose of intravenous methylprednisolone (IVMP), with marked improvement of the visual deficit.

### Case 3

A 54-year-old woman was diagnosed with MS in 1993. She remained clinically stable without any therapy up to 2021. On February 27, 2021, 3 days after the first ChAdOx1 nCoV-19 dose, the patient developed hypoesthesia below the T6 level. She underwent a new MRI showing one enhancing lesion in the spinal cord (Figure 1C). She was treated with IVMP with complete recovery.

### Case 4

A 66-year-old woman received the first dose of ChAdOx1 nCoV-19 on April 11, 2021 and, 1 week later, complained visual disturbance and postural instability on the right limbs. A brain MRI on May 4 showed multiple white matter lesions, four of them enhancing in the left paratrigonal and periventricular white matter (Figure 1D). Her CSF showed oligoclonal bands. Diagnosis of MS (7) was made, and she was treated with IVMP with partial improvement.

### Case 5

In 2019 a 42-year-old woman experienced a progressive weakness on the right side of her body. After an MRI scan performed in February 2020, showing multiple lesions with dissemination in space and time, she started treatment with Ocrelizumab on May 8, 2020. She received the first dose of mRNA-1273 vaccine on March 22, 2021. Two weeks later, she experienced slight weakness of the left upper limb. On April 19, 2021, she received the booster, and after 3 days, her follow-up MRI showed an enhancing brain lesion in the right corona radiata (Figure 1E).

### Case 6

A 57-year-old man had a diagnosis of MS in 2001. He was treated initially with injectables, then with teriflunomide, and, in 2015, with mitoxantrone. Since then, he remained clinically and radiologically stable without any treatment. On May 11, 2021, he received the booster of mRNA-1273 vaccine. Two weeks later, he experienced a severe motor deficit in both legs that made him bed bound. He was admitted to hospital where he underwent an MRI on June 7, 2021, showing an enhancing pontine lesion (Figure 1F). He was treated with IVMP with only partial recovery.

### Case 7

A 49-year-old woman was diagnosed with MS in November 2013. She has been on treatment with dimethyl fumarate (DMF) since July 2014, with clinical and radiological stability. On April 1, 2021, she underwent a brain and spinal cord MRI scan, which was stable. On April 8, she received her first BNT162b2 dose of vaccine. Five days after, she developed numbness on the left hand and left side of her head. On April 20, she underwent a new scan, which detected a periventricular lesion and a spinal lesion at C3 level, both enhancing (Figure 1G). She was treated with IVMP with almost complete recovery.

### Case 8

In 2014, after the onset of hypoesthesia on his left side, a 39-year-old man underwent an MRI scan, which showed multiple lesions on brain and spinal cord. He started treatment with injectables switched to DMF in 2017. After almost 3 years of clinical and radiological stability, on April 27, 2021, he received his first dose of BNT162b2 vaccine, followed, 10 days later, by the onset of paraesthesia on his left leg. He underwent an MRI scan on May 13 that showed three new lesions, two of which were enhancing in the left parietal lobe and in the periventricular white matter (Figure 1H). He was treated with oral steroids with partial recovery.

### Case 9

A 39-year-old woman suffered from her first clinical episode in August 2019 with a complete recovery. A diagnosis of clinically isolated syndrome was made, and she was monitored by serial MRI that confirmed a radiological stability up to January 2021. On April 29, she received her first dose of BNT162b2 vaccine followed, 3 days later, by dysesthesia on her right hand and foot. A scan performed on May 11, 2021 showed a new enhancing lesion in the mesencephalon (Figure 1I). She was treated with IV methylprednisolone with a good recovery. A diagnosis of MS was made, and a DMT was planned.

### Case 10

A 60-year-old female patient received a diagnosis of MS in 1998. In 2001, she started treatment with injectables switched to DMF in 2015. She was clinically and radiologically stable for 6 years. In April 2021, she performed the first BNT162b2 dose of vaccine presenting few days later with fatigue and numbness in both legs. A scan was performed, and one enhancing brain lesion was detected in the left periventricular white matter (Figure 1L).

### Case 11

A 30-year-old woman was diagnosed with MS in 2018, after a clinical onset with optic neuritis and MRI suggestive of dissemination in space and time. She was treated with DMF between September 2018 and August 2020 and then she started Cladribine. A baseline MRI at the end of October 2020 was stable. She received the BNT162b2 booster on April 8, 2021. Twenty days later, she complained of a language disturbance. A brain MRI performed on June 3, 2021 revealed the presence of two enhancing brain lesions, one in the right corona radiata and one with conspicuous oedema in the left centrum semiovale (Figure 1M).

### Case 12

A 58-year-old woman was diagnosed with MS in August 2000. She was treated with injectables and then, in 2018, with DMF that was stopped after 1 year for lymphopenia. She performed an MRI scan in February 2020 that was stable. She had her first BNT162b2 dose on March 26, 2021. Three days later, she complained headache, balance disturbances, urinary incontinence, difficulties in walking, and dysphagia. She performed an MRI on May 27, 2021 that showed a new area with ring enhancement in the white matter of the left frontal lobe (Figure 1N). She started IVMP with benefit.

### Case 13

A 34-year-old woman developed numbness and hyposthenia on her right hand in February 2021. An MRI scan showed multiple lesions and one enhancing cord lesion at C3 level. Diagnosis of MS was made. She was treated with IVMP with almost complete recovery. A treatment with Ocrelizumab was planned. On May 18, she received the BNT162b booster. Four days later, she complained of neck pain and hypoesthesia on her right arm. She performed an MRI scan on June 7 showing three brain enhancing lesions (one right posterior paraventricular and two in the left periventricular white matter) and a new unenhanced lesion on spinal cord (Figure 1O).

### Case 14

A 35-year-old woman received a diagnosis of MS in the 2005. She was treated with injectables, and in February 2019, she started DMF. She remained clinically and radiologically stable until May 24, 2021, when she received the BNT162b2 booster. The day after the vaccination, she developed paraesthesia on the left side of the body. She underwent a scan 13 days later, which showed three enhancing lesions in the left temporal lobe and left centrum semiovale (Figure 1P).

### Case 15

A 54-year-old man was diagnosed with MS in 2003. He was treated with injectables and switched to teriflunomide in November 2020. He was clinically stable and without new lesions on MRI performed on February 25, 2021. On April 7, 2021, 1 week after the first dose of BNT162b2 vaccine (March 31, 2021), he developed a right hemiparesis. A brain scan showed two ring-enhancing lesions located in the left periventricular white matter (Figure 1Q). IVMP was administered with full recovery. He received the BNT162b2/Pfizer-BioNTech booster on May 11, 2021. without any further medical problem.

### Case 16

A 37-year-old man was diagnosed with MS in 2019. In April 2020, he started DMF with clinical stability. On June 4, 2021, he had the BNT162b2 booster. On June 15, the patient presented with weakness on his right limbs. On June 24, he underwent a brain MRI that, compared with a previous routine scan of May 20, 2021, showed a new tumefactive contrast-enhancing lesion in the left fronto-parietal white matter (Figure 1R). The patient was treated with IVMP with partial recovery.

## Discussion

There have been few cases reported of neurological complications associated with COVID-19 vaccination. These include cases of transverse myelitis (8), of Bell's palsy (9), of unusual variant of Guillain-Barre syndrome, and of cerebral venous sinus thrombosis (10, 11). In MS, there are suggestions of an unchanged rate of relapse in vaccinated, when compared to non-vaccinated, patients following the vaccination (12). However, this latter finding has not been supported by radiological evidence of disease activity and the period of observation was limited.

Only two cases of acute relapse after COVID-19 vaccination have been reported so far (13, 14), both having a good outcome.

Here, we describe a series of 16 patients with MS relapses occurring from 3 days to 3 weeks after their COVID-19 vaccination, between March and June 2021. During this period, at least 2500 patients with MS accessed the four MS Centres. During this period, a total of 69 verified (i.e., treated with high dose IV steroids) relapses were observed in the Centers, while 52 relapses were measured in the preceding 4 months. Although seasonal variation in relapse rate associated with monthly hours of sunshine should be taken into account (15), an increase in the total number of relapses was observed during the SARS-COV-2 vaccination campaign.

Out of 16 cases, 3 received a diagnosis of MS after COVID-19 vaccination; the remaining 13 had already a diagnosis of MS made from few months to several years before the vaccine administration. Nine patients were on DMTs; four patients were no longer on DMTs, although they had used them in the past, and were clinically and radiologically stable. Disease reactivation is reported after both the first vaccine administration (*n* = 10) and the booster (*n* = 6). All patients had evidence of radiological activity on MRI to support the relapse diagnosis. Age, sex, and level of disability reflect what may be expected for a relapsing MS cohort. The characteristics of the enhancing lesions varied from small to large lesions, in both the brain and the spinal cord.

The role of vaccines on the risk of developing MS and MS relapses remains to be elucidated, with no sufficient data to support or refute an association between the development of MS and the antiviral vaccinations (16, 17). Therefore, currently, there are no contraindications for vaccination in patients with MS, with the only exception regarding live-attenuated vaccines that are contraindicated for MS patients who receive immunosuppressive or immunomodulating treatments. Unless the risk of infection outweighs the risk of adverse reactions induced by the vaccine, MS relapses are not a contraindication for vaccination, but they are a reason to delay vaccination until remission (17).

Although the evidence of an association between vaccination and MS activity is still debated (18), a link between them has been suggested, within the first 30 days after immunization, given the possibility of vaccines to accelerate the transition from subclinical to clinical disease through a stimulation of the immune system (19). In a previous case series, we have looked into the safety of receiving the influenza vaccine in MS patients by clinical and MRI studies, adding a note of caution in those subjects with evidence of recent disease activity (20).

The exact mechanisms through which autoimmune reactions can be triggered by vaccination are not fully understood, although they probably vary according to the type of vaccine and individual genetic susceptibility (21, 22).

Immunological studies have shown that coordinated interactions between T and B lymphocytes of the adaptive immune system are necessary for the successful generation of immunological memory and the production of neutralizing antibodies following recognition of antigens by the innate immune cells (3). However, the T/B cell interaction may be altered in MS even in the absence of DMTs (23, 24). In addition, new technologies currently used for mounting an immune response to the COVID-19 vaccine, such as mRNA vaccines, have not been tested in populations suffering from autoimmune conditions before the vaccination campaign.

Although the clinical cases described here experienced neurological symptoms that were temporally associated with administration of the vaccine, causality cannot be assumed. Indeed, it cannot be disentangled whether radiologically confirmed relapses occurring after vaccination are triggered by the vaccination-induced inflammatory state or are relapses that would have happened anyway, independently from vaccination.

After the authorization of vaccines against SARS-CoV-2 in Italy, MS patients were prioritized for vaccination starting in March 2021. The availability of COVID-19 vaccines met the willingness of approximately 80% of European MS patients to receive vaccination (ref). The greatest interest in vaccination was observed in older patients and in those with comorbidities (25). This evidence is reflected in our case series, where mean age was 46.7 ± 10.3 and median EDSS score was 2.5. Immunosenescence increases the risk of adverse events in older adults. Beyond reduced response to vaccines, changes that take place in the immune system with aging generally result in higher susceptibility to infections and prevalence of autoimmunity (26), factors that can precipitate reactivations in MS patients. During mass immunization campaign, such as that occurred in France between 1995 and 1997, several cases of MS were reported a few weeks after HBV vaccination, suggesting that vaccine may accelerate the transition from subclinical to clinical disease (27). However, two subsequent case-control studies showed a non-significant increase in risk of developing MS following the HBV vaccine (ref). Therefore, conclusions derived from case reports and case series are not free from biases and should not influence vaccine hesitancy (28).

Adverse events of vaccination can occur in rare cases, but benefits generally outweigh adverse effects, given that acute infections may have dangerous consequences (29). Indeed, patients with MS have an increased mortality risk from COVID-19, especially if older and with significant disability and/or comorbidities (30).

## Conclusion

The elevated number of MS patients with relapse after COVID-19 vaccine coming to our observation during a relative short period of time suggests the need for robust post-vaccination surveillance in patients with MS. Large prospective controlled studies are required to estimate the frequency of MS relapses, both clinically and MRI proved, which might occur during the post-vaccine period when a new COVID-19 vaccination program will be planned.

## Data Availability Statement

The original contributions presented in the study are included in the article/supplementary material, further inquiries can be directed to the corresponding authors.

## Ethics Statement

Written informed consent was obtained from the individual(s) for the publication of any potentially identifiable images or data included in this article.

## Author Contributions

CP and RN wrote the manuscript with support from EB, AI, LT, FM, LP, GD, VP, and VT. CP and VR conceived of the presented idea. All authors have collected the data.

## Conflict of Interest

FM has received consulting fees, speaker honoraria, and/or travel grants from Biogen, Sanofi Genzyme, Novartis, and Roche; GD served on scientific advisory boards for Merck, Sanofi-Genzyme, and Roche, and has received travel and/or speaker honoraria from Merck, Roche, Teva, Biogen, Novartis, and Sanofi-Genzyme. LP has received consulting fees from Celgene, Biogen, and Novartis; speaker honoraria and/or travel grants from Biogen, Genzyme, Merck Serono, Novartis, Roche, and Teva; research grants from the Italian MS Society (Associazione Italiana Sclerosi Multipla) and Genzyme. VT has received consulting fees and/or travel grants and/or research grants from Bristol Myer Squibb, Biogen, Novartis, Sanofi Genzyme, Merck Serono, and Roche; research grants from the Italian MS Society (Associazione Italiana Sclerosi Multipla) and the MS Society UK, and from the Italian Ministry of Health. CP has served on scientific advisory boards for Novartis, Merck, Biogen, Sanofi, Genzyme, Teva, and Actelion; received funding for travel and speaker honoraria from Biogen, Teva, Sanofi Genzyme, Actelion, and Novartis; received research support from Biogen, Teva, Novartis, and Genzyme. The remaining authors declare that the research was conducted in the absence of any commercial or financial relationships that could be construed as a potential conflict of interest.

## Publisher's Note

All claims expressed in this article are solely those of the authors and do not necessarily represent those of their affiliated organizations, or those of the publisher, the editors and the reviewers. Any product that may be evaluated in this article, or claim that may be made by its manufacturer, is not guaranteed or endorsed by the publisher.

## References

[1] Persson R Lee S Yood MU Wagner Usn Mc CM Minton N Niemcryk S. Infections in patients diagnosed with multiple sclerosis: a multi-database study. Mult Scler Relat Disord. (2020) 41:101982. 10.1016/j.msard.2020.101982 32070858

[2] Cahill JF Izzo A Garg N. Immunization in patients with multiple sclerosis. Neurological Bulletin. (2010) 2:17–21. 10.7191/neurol_bull.2010.1020

[3] Coyle PK Gocke A Vignos M Newsome SD. Vaccine considerations for multiple sclerosis in the COVID-19 Era. Adv Ther. (2021) 38:3550–88. 10.1007/s12325-021-01761-3 34075554 PMC8169434

[4] Mailand MT Frederiksen JL. Vaccines and multiple sclerosis: a systematic review. J Neurol. (2017) 264:1035–50. 10.1007/s00415-016-8263-4 27604618

[5] Schlake T Thess A Fotin-Mleczek M Kallen KJ. Developing mRNA-vaccine technologies. RNA Biol. (2012) 9:1319–30. 10.4161/rna.22269 23064118 PMC3597572

[6] Du L Zhao G Lin Y Sui H Chan C Ma S. Intranasal vaccination of recombinant adeno-associated virus encoding receptor-binding domain of severe acute respiratory syndrome coronavirus (SARS-CoV) spike protein induces strong mucosal immune responses and provides long-term protection against SARS-CoV infection. J Immunol. (2008) 180:948–56. 10.4049/jimmunol.180.2.948 18178835 PMC2603051

[7] Thompson AJ Banwell BL Barkhof F Carroll WM Coetzee T Comi G. Diagnosis of multiple sclerosis: 2017 revisions of the McDonald criteria. Lancet Neurol. (2018) 17:162–73. 10.1016/S1474-4422(17)30470-2 29275977

[8] Knoll MD Wonodi C. Oxford-AstraZeneca COVID-19 vaccine efficacy. Lancet. (2021) 397:72–4. 10.1016/S0140-6736(20)32623-4 33306990 PMC7832220

[9] Goss AL Samudralwar RD Das RR Nath A. ANA investigates: neurological complications of COVID-19 vaccines. Ann Neurol. (2021) 89:856–7. 10.1002/ana.26065 33710649 PMC8250888

[10] Allen CM Ramsamy S Tarr AW Tighe PJ Irving WL Tanasescu R. Guillain-Barré syndrome variant occurring after SARS-CoV-2 vaccination. Ann Neurol. (2021) 90:315–8. 10.1002/ana.26144 34114269

[11] European Medicines Agency EMA. AstraZeneca's COVID-19 Vaccine: *EMA Finds Possible Link to Very Rare Cases of Unusual Blood Clots With Low Blood Platelets* (2021). Available online at: https://anmj.org.au/update-astrazeneca-covid-19-vaccine-blood-clots-with-low-platelet-counts/ (accessed April 2021).

[12] Achiron A Dolev M Menascu S Zohar DN Dreyer-Alster S Miron S. COVID-19 vaccination in patients with multiple sclerosis: what we have learnt by February 2021. Mult Scler. (2021) 27:864–70. 10.1177/13524585211003476 33856242 PMC8114441

[13] Etemadifar M Sigari AA Sedaghat N Salari M Nouri H. Acute relapse and poor immunization following COVID-19 vaccination in a rituximab-treated multiple sclerosis patient. Hum Vaccin Immunother. (2021) 20:1–3. 10.1080/21645515.2021.1928463 34015240 PMC8437516

[14] Maniscalco GT Manzo V Di Battista ME Salvatore S Moreggia O Scavone C. Severe multiple sclerosis relapse after COVID-19 vaccination: a case report. Front Neurol. (2021) 12:721502. 10.3389/fneur.2021.721502 34447349 PMC8382847

[15] Harding K Tilling K MacIver C Willis M Joseph F Ingram G. Seasonal variation in multiple sclerosis relapse. Neurol. (2017) 264:1059–67. 10.1007/s00415-017-8485-0 28424900 PMC5486559

[16] Lebrun C Vukusic S French group for recommendations in multiple sclerosis (France4MS) The Société Francophone De La Sclérose En Plaques (SFSEP). Immunization and multiple sclerosis: recommendations from the French multiple sclerosis society. Mult Scler Relat Disord. (2019) 31:173–88. 10.1016/j.msard.2019.04.004 31159998

[17] Farez MF Correale J Armstrong MJ Rae-Grant A Gloss D Donley D. Practice guideline update summary: vaccine-preventable infections and immunization in multiple sclerosis: report of the guideline development, dissemination, and implementation subcommittee of the american academy of neurology. Neurology. (2019) 93:584–94. 10.1212/WNL.0000000000008157 31462584

[18] Zrzavy T Kollaritsch H Rommer PS Boxberger N Loebermann M Wimmer I. Vaccination in multiple sclerosis: friend or foe? Front Immunol. (2019) 10:1883. 10.3389/fimmu.2019.01883 31440255 PMC6693409

[19] Langer-Gould A Qian L Tartof SY Brara SM Jacobsen SJ Beaber BE. Vaccines and the risk of multiple sclerosis and other central nervous system demyelinating diseases. JAMA Neurol. (2014) 71:1506–13. 10.1001/jamaneurol.2014.2633 25329096

[20] Salvetti M Pisani A Bastianello S Millefiorini E Buttinelli C Pozzilli C. Clinical and MRI assessment of disease activity in patients with multiple sclerosis after influenza vaccination. J Neurol. (1995) 242:143–6. 10.1007/BF00936886 7751856

[21] Kivity S Agmon-Levin N Blank M Shoenfeld Y. Infections and autoimmunity: Friends or foes? Trends Immunol. (2009) 30:409–14. 10.1016/j.it.2009.05.005 19643667

[22] Chen RT Pless R Destefano F. Epidemiology of autoimmune reactions induced by vaccination. J Autoimmun. (2001) 16:309–18. 10.1006/jaut.2000.0491 11334497

[23] Hartmut Wekerle. B cells in multiple sclerosis. Autoimmunity. (2017) 50:57–60. 10.1080/08916934.2017.1281914 28166681

[24] Severson C Hafler DA. T-Cells in Multiple Sclerosis. Results Probl Cell Differ. Berlin; Heidelberg: Springer-Verlag (2009). 10.1007/400_2009_12 19582415

[25] Serrazina F Pinho AS Cabral G Salavisa M Correia AS. Willingness to be vaccinated against COVID-19: an exploratory online survey in a Portuguese cohort of multiple sclerosis patients. Mult Scler Relat Disord. (2021) 51:102880. 10.1016/j.msard.2021.102880 33740481 PMC7932878

[26] Dema M Eixarch H Villar LM Montalban X Espejo C. Review. Immunosenescence in multiple sclerosis: the identification of new therapeutic targets. Autoimmunity Rev. (2021) 20:102893. 10.1016/j.autrev.2021.102893 34237417

[27] Ascherio A Zhang SM Hernán MA Olek MJ Coplan PM Brodovicz K. Hepatitis B vaccination and the risk of multiple sclerosis. N Engl J Med. (2001) 344:327–32. 10.1056/NEJM200102013440502 11172163

[28] Diem L Friedli C Chan A Salmen A Hoepner R. Vaccine hesitancy in patients with multiple sclerosis: preparing for SARS-Cov 2 vaccination challenge. Neurol Neuroimmunol Neuroinflamm. (2021) 8:e991. 10.1212/NXI.0000000000000991 33811158 PMC8018793

[29] Sirbu CA Florea AA Ghinescu MC Docu-Axelerad A Sirbu AM Bratu OG. Vaccination in multiple sclerosis - challenging practices (Review). Exp Ther Med. (2020) 20:217. 10.3892/etm.2020.9347 33149781 PMC7604740

[30] Barzegar M Mirmosayyeb O Gajarzadeh M Afshari-Safavi A Nehzat N Vaheb S. COVID-19 among patients with multiple sclerosis: a systematic review. Neurol Neuroimmunol Neuroinflamm. (2021) 8:e1001. 10.1212/NXI.0000000000001001 34016734 PMC8142838

